# Supplementary material for: Adverse PFAS effects on mouse oocyte in vitro maturation are associated with carbon‐chain length and inclusion of a sulfonate group
Source: Cell Prolif. 2022 Oct 27;56(2):e13353. doi: 10.1111/cpr.13353 (PMC9890540; doi:10.1111/cpr.13353)
Supplement: Supplementary file 1 — Figure S1 The toxic effects of PFHxS on mouse oocyte in vitro maturation are positively correlated with the concentrations. (A) Average GVBD rates in oocytes exposed to different concentrations of PFHxS. (B) Average PBE rates in oocytes exposed to different concentrations of PFHxS. A total of 175 oocytes in the control group, 68 oocytes in the 300 μM group, 179 oocytes in the 600 μM group, and 68 oocytes in the 1200 μM group were analysed to calculate GVBD and PBE rates. (C) PFHxS‐induced size ratio increase was positively correlated with concentrations. A total of 40 oocytes in the control group, 58 oocytes in the 300 μM group, 63 oocytes in the 600 μM group, and 16 oocytes in the 1200 μM group were measured to calculate the size ratios. Data in bar chart were presented as mean ± SEM. All groups had at least 3 independent replicates. Figure S2. PFCA at 600 μM showed no effects on mouse oocyte in vitro maturation. (A) Representative images show GVBD and PBE of four treatment groups (untreated, 600 μM PFBA, 600 μM PFHxA, and 600 μM PFOA). The red arrows indicate oocytes that retained their germinal vesicles after 2 h of culture. The yellow asterisks indicate oocytes that did not extrude a PB after 14 h of culture. Scale bar, 100 μm. (B) The rates of GVBD in the control and PFCA‐treated groups. (C) The rates of PBE in the control and PFCA‐treated groups. A total of 107 oocytes in the control group, 117 oocytes in the PFBA‐treated group, 124 oocytes in the PFHxA‐treated group, and 144 oocytes in the PFOA‐treated group were analysed to calculate the GVBD and PBE rates. (D) The size ratios of PBs to oocytes in the control and PFCA‐treated groups. A total of 41 oocytes in the control group, 52 oocytes in the PFBA‐treated group, 56 oocytes in the PFHxA‐treated group, and 63 oocytes in the PFOA‐treated group were measured to calculate the size ratios. Data in bar chart were presented as mean ± SEM. All groups had at least three independent groups. Figure S3. Time‐lapse brig [file CPR-56-e13353-s001.docx]

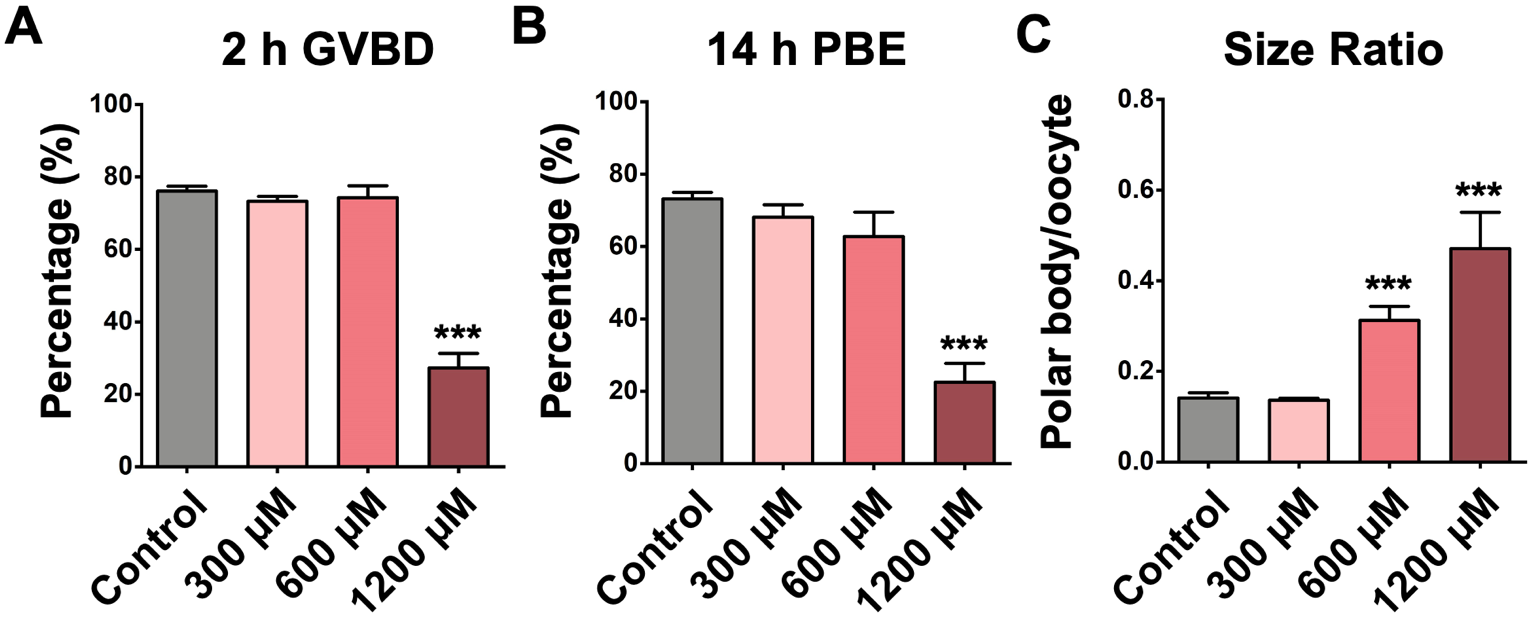


**Supplemental Figure 1.** The toxic effects of PFHxS on mouse oocyte *in vitro* maturation are positively correlated with the concentrations. (A) Average GVBD rates in oocytes exposed to different concentrations of PFHxS. (B) Average PBE rates in oocytes exposed to different concentrations of PFHxS. A total of 175 oocytes in the control group, 68 oocytes in the 300 µM group, 179 oocytes in the 600 µM group, and 68 oocytes in the 1200 µM group were analyzed to calculate GVBD and PBE rates. (C) PFHxS-induced size ratio increase was positively correlated with concentrations. A total of 40 oocytes in the control group, 58 oocytes in the 300 µM group, 63 oocytes in the 600 µM group, and 16 oocytes in the 1200 µM group were measured to calculate the size ratios. Data in bar chart were presented as mean ± SEM. All groups had at least 3 independent replicates.


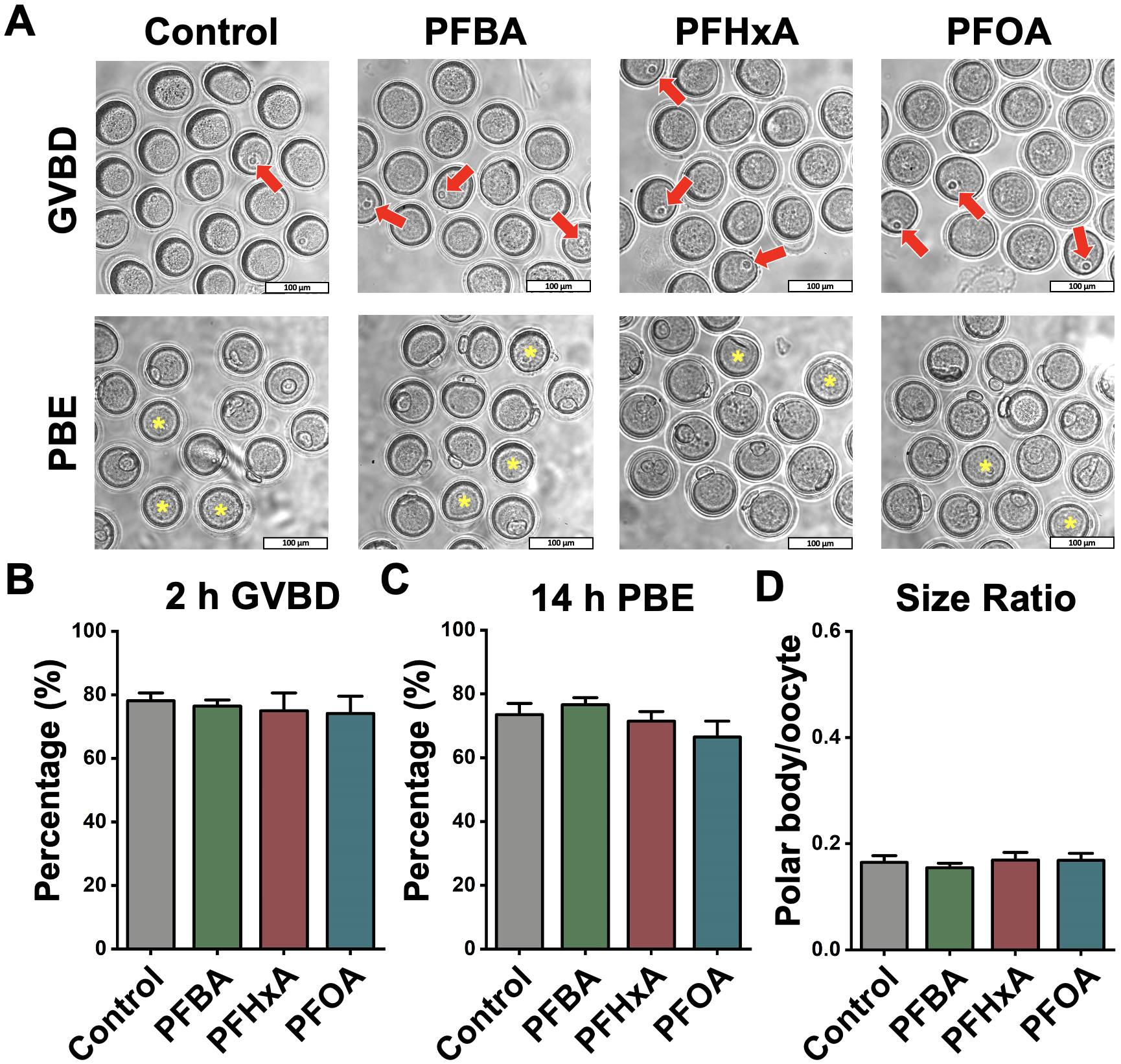


**Supplemental Figure 2.** PFCA at 600 µM showed no effects on mouse oocyte *in vitro* maturation. (A) Representative images show GVBD and PBE of four treatment groups (untreated, 600 μM PFBA, 600 μM PFHxA, and 600 μM PFOA). The red arrows indicate oocytes that retained their germinal vesicles after 2 hours of culture. The yellow asterisks indicate oocytes that did not extrude a PB after 14 hours of culture. Scale bar, 100 μm. (B) The rates of GVBD in the control and PFCA-treated groups. (C) The rates of PBE in the control and PFCA-treated groups. A total of 107 oocytes in the control group, 117 oocytes in the PFBA-treated group, 124 oocytes in the PFHxA-treated group, and 144 oocytes in the PFOA-treated group were analyzed to calculate the GVBD and PBE rates. (D) The size ratios of PBs to oocytes in the control and PFCA-treated groups. A total of 41 oocytes in the control group, 52 oocytes in the PFBA-treated group, 56 oocytes in the PFHxA-treated group, and 63 oocytes in the PFOA-treated group were measured to calculate the size ratios. Data in bar chart were presented as mean ± SEM. All groups had at least 3 independent groups.


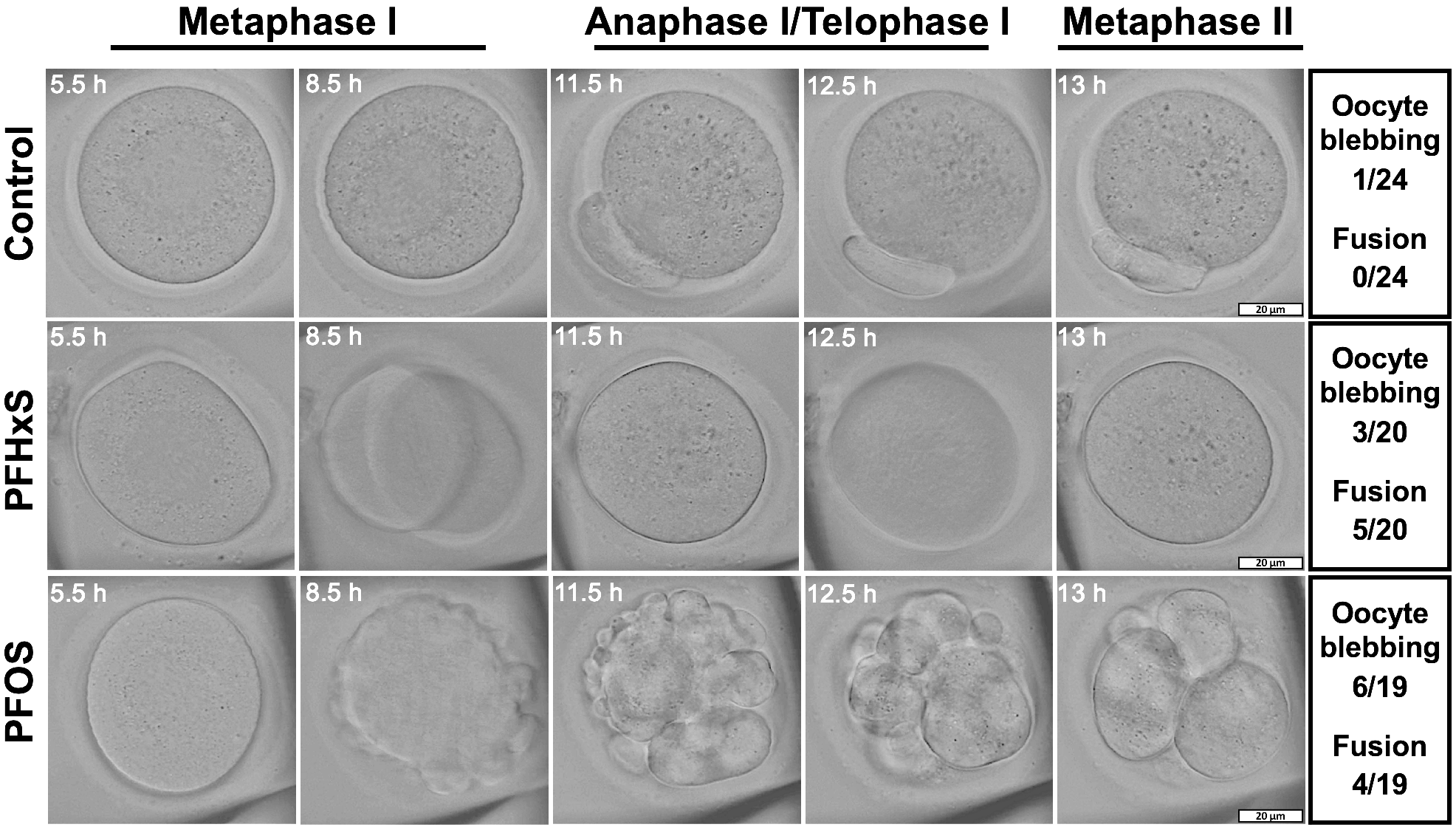


**Supplemental Figure 3.** Time-lapse bright-field images showing oocyte maturation process in the control, 600 µM PFHxS, and 600 µM PFOS groups. The oocyte in the control group extruded normal first polar body (PB) at anaphase I/telophase I. However, the oocyte in the PFHxS group extruded its large polar body prior to its resorption (oocyte and PB failure, cytokinesis failure). The oocyte in PFOS group underwent severe cytoplasmic blebbing followed cytokinesis defects. The table on the right listed the numbers of oocytes that experienced blebbing and fusion. Scale bar, 20 µm.
